# Effect of vitamin D supplementation on the incidence and prognosis of depression: An updated meta-analysis based on randomized controlled trials

**DOI:** 10.3389/fpubh.2022.903547

**Published:** 2022-08-01

**Authors:** Fei Xie, Tongmin Huang, Dandi Lou, Rongrong Fu, Chaoxiong Ni, Jiaze Hong, Lingyan Ruan

**Affiliations:** ^1^Department of Endocrinology, Ningbo Yinzhou No. 2 Hospital, Ningbo, China; ^2^The Second Clinical Medical College, Zhejiang Chinese Medical University, Hangzhou, China; ^3^The First Clinical Medical College, Zhejiang Chinese Medical University, Hangzhou, China; ^4^Department of Nephrology, QingChun Hospital of Zhejiang Province, Hangzhou, China

**Keywords:** depression, vitamin D supplementation, incidence, prognosis, meta-analysis

## Abstract

**Background:**

There have been several controversies about the correlation between vitamin D and depression. This study aimed to investigate the relationship between vitamin D supplementation and the incidence and prognosis of depression and to analyze the latent effects of subgroups including population and supplement strategy.

**Methods:**

A systematic search for articles before July 2021 in databases (PubMed, EMBASE, Web of Science, and the Cochrane Library) was conducted to investigate the effect of vitamin D supplementation on the incidence and prognosis of depression.

**Results:**

This meta-analysis included 29 studies with 4,504 participants, indicating that the use of vitamin D was beneficial to a decline in the incidence of depression (SMD: −0.23) and improvement of depression treatment (SMD: −0.92). Subgroup analysis revealed that people with low vitamin D levels (<50 nmol/L) and females could notably benefit from vitamin D in both prevention and treatment of depression. The effects of vitamin D with a daily supplementary dose of >2,800 IU and intervention duration of ≥8 weeks were considered significant in both prevention and treatment analyses. Intervention duration ≤8 weeks was recognized as effective in the treatment group.

**Conclusion:**

Our results demonstrate that vitamin D has a beneficial impact on both the incidence and the prognosis of depression. Whether suffering from depression or not, individuals with low vitamin D levels, dose >2,800 IU, intervention duration ≥8 weeks, and all females are most likely to benefit from vitamin D supplementation.

## Introduction

Depression is a worldwide health issue for people that commonly coexists with other debilitating chronic illnesses. According to the World Health Organization, approximately 350 million people worldwide suffer from depression. It has been regarded as the leading cause of significant disability and mortality (1–3). Despite decades of intensive neurobiologically focused psychiatric study, the etiology of depression still remains a mystery. Classical antidepressants mainly increase the concentration of monoamines in the synaptic space by blocking the reuptake of serotonin and norepinephrine at presynaptic nerve terminals (4). However, approximately half of the patients fail to respond to the first-line treatment of antidepressants (5). Besides, the side effects of antidepressants are hard to be ignored. Hence, finding other clinical programs to optimize the treatment of depression appears extremely urgent.

Vitamin D has been investigated to be vital in the normal development and functionalization of the brain. The insufficient and deficiency of vitamin D are related to neurological disorders (6, 7). Also, the correlation between low-level vitamin D and depression has been proved (8). Epidemiological surveys demonstrated that the incidence of depression was 8–14% higher in people with vitamin D deficiency (9–11). Therefore, the implementation of vitamin D in anti-depression treatment has raised awareness among healthcare professionals. Compared with antidepressants, vitamin D is still believed by some to have more favorable safety and better patient compliance despite the adverse effect caused by excessive vitamin D. Nevertheless, the related results still remain controversial.

Previously, a meta-analysis exploring the effects of vitamin D supplements in the management of depression showed that vitamin D supplementation was therapeutically effective in relieving the symptoms of depression (2). However, in two subsequent meta-analyses, there was no evidence that vitamin D supplementation was always desirable in relieving depressive symptoms in people with different health problems (12). A recent meta-analysis demonstrated that vitamin D supplementation could reduce the occurrence of negative emotions, including depression and anxiety, to some extent (13). However, due to the small number of studies, absence of clear distinction between the prevention and treatment consequences of vitamin D for depression, and no clear requirements for the placebo control of the trial, these findings should be interpreted with caution. Based on the shortcomings above and in the hope of providing some guiding significance for clinical application, we intend to review the relevant randomized controlled trials (RCTs) and conduct an updated meta-analysis to investigate the effect of vitamin D supplementation on the incidence and prognosis of depression.

## Methods

### Search strategy

According to the preferred reporting items for Systematic Review and Meta-Analysis (PRISMA) 2015 (14), two authors independently ran a systematic search of PubMed, EMBASE, Web of Science, and the Cochrane Library, and covered all potentially relevant articles to appraise the effect of vitamin D supplementation on the incidence and prognosis of depression. And the time scope of the literature search was limited from the establishment of the database to July 2021. Literature retrieval was carried out through the random combinations of the following search terms: “vitamin D” OR “vitamin D supplementation” OR “25-hydroxyvitamin D” OR “25-hydroxyvitamin D supplementation” OR “25(OH)D” OR “cholecalciferol” AND “depression” OR “depressive” OR “negative emotion” OR “major depressive disorder” AND “placebo.” The references in the primary articles and relevant reviews were also reviewed manually to avoid the omission of any potentially relevant research.

### Inclusion and exclusion criteria

The inclusion criteria were as follows: (1) RCTs were included in the study; (2) studies in which the experimental groups were supplied with vitamin D supplementation, and the control groups were in absence of vitamin D or only supplemented with placebo in both at baseline and terminal of the intervention; (3) no psychological symptoms in depression or a diagnosis of depression was in the population (two groups of the population could not be mixed into the same study); (4) the participants in each group were categorized under the depression assessment scale in both at baseline and terminal of the intervention, based on their psychological symptoms, to assess the impact of vitamin D on the incidence and prognosis of depression.

The exclusion criteria were as follows: (1) study that was not published in English; (2) study in which the effects of vitamin D supplementation on the baseline and terminal of were only compared in the experimental groups without a control group; (3) study that was just a study protocol or without outcome reported; (4) the intervention contained nutritional supplements/medications; (5) study that unable to obtain the full text or extract available data; (6) study that was a duplicate publication (the latest published or most complete studies would be selected for inclusion).

### Data extraction

The retrieved articles were independently screened by two authors, and the data were extracted following a pre-designed data extraction table. Any divergence was resolved through a third-party discussion. Data extracted from each study included: author; year of publication; country; follow up duration; participants' characteristics (e.g., mean age, sample size, body mass index (BMI), serum level of 25(OH)D at baseline and terminal); the dose of vitamin D supplementation; trial registration number; the mean and standard deviation in participants' depression assessment scale scores at baseline, terminal, and the difference between. Different depression assessment scales that were used for the evaluation of the psychological symptoms in different studies include the Beck Depression Inventory (BDI), BDI-II, Hospital Anxiety and Depression Scale (HADS), Hamilton Depression Rating Scale (HAM-D), etc. When multiple depression assessment scales were used in the same study, priority was given to the more well-known and the more commonly used ones (13).

### Quality evaluation and outcome measures

The Cochrane Collaborative Risk of Bias Assessment Tool was used to assess the potential risk of bias in RCTs (15). It contains six domains: sequence generation, allocation concealment, blinding, incomplete outcome data, selective outcome reporting, and other sources of bias. The risk was classified into three levels: high risk, unclear risk, and low risk.

The efficacy outcome measure of this meta-analysis was the differences in the depression scores from baseline to terminal both in the experimental groups and the control groups, which were assessed by multiple depression assessment scales.

### Statistical analysis

By using Revman 5.3 software, each study on the total differences in changes in depressive symptoms between vitamin D groups and control groups was all combined to evaluate the overall effect of vitamin D supplementation on the incidence and prognosis of depression. Through the data extracted from each study, for continuous variables, the effect size was calculated as standardized mean differences with a 95% confidence interval (CI). For the entire sample, the heterogeneity test was estimated using the Cochran chi-square test and quantified using the inconsistency test (I^2^) (16). Considering the differences in the type and dose of vitamin D supplements, a random-effects model was applied. All probabilities (*p*-values) of data were two-sided, with *p* < 0.05 regarded as being statistically significant.

The potential variables leading to sources of heterogeneity were investigated *via* the subgroup analysis. The potential variables included: participants' level of serum 25(OH)D, the dose of vitamin D supplementation, the depression assessment scale that was well-known and commonly used, BMI of participants, mean age of participants, participant's gender, duration of intervention. When included studies ≥10, the publication bias would be assessed using a funnel plot (17). In addition, the software was also used for the sensitivity analysis, which was conducted to estimate whether it would affect the pooled effect by excluding each study successively.

## Results

### Study selection

**Figure 1:**
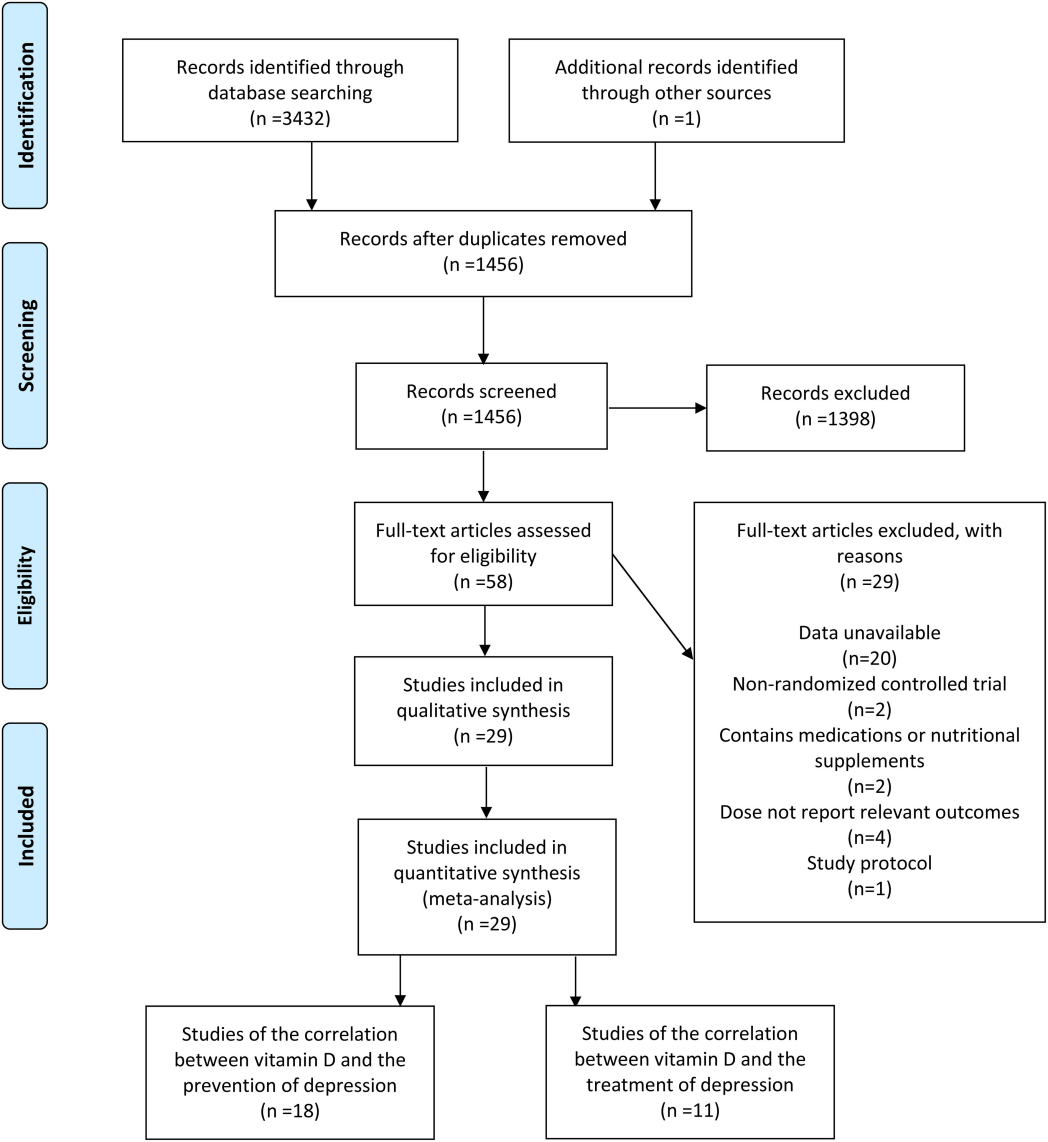
Flow diagram describing inclusion and exclusion criteria.

A total of 3,432 relevant studies were reviewed after the database search. In addition, one study was identified through other origins. After removing 1,977 duplicate studies, 1,398 studies were ruled out through the screening of their title and abstract. Additionally, at the back of the full-text review and evaluation of the remaining 58 studies, one study protocol was excluded, and 20 were excluded due to unavailable data. Furthermore, two studies were excluded because of non-RCT, four articles were excluded because they failed to report relevant outcomes, and two articles containing medications or nutritional supplements were also excluded. Ultimately, in accordance with inclusion and exclusion criteria, 29 eligible studies (7, 18–45) were included (Figure 1), 18 studies (18–35) for the correlation between vitamin D and the incidence of depression, and 11 studies (7, 36–45) for the correlation between vitamin D and the development of depression.

### Study characteristics

The basic characteristics of 18 studies illustrating the correlation between vitamin D and the occurrence of depression are displayed in Table 1 and Supplementary Table 1. The locations of the studies were classified as North America, Europe, Asia, and Oceania. Participants in these studies ranged in age from 13 to 85 years old with 2,111 cases in the experimental groups and 2,147 cases in the control groups. Dosage of vitamin D ranged from 200 IU per day to about 10,714 IU per day. The follow-up duration ranged from 6 to 144 weeks. The basic characteristics of 11 studies demonstrating the correlation between vitamin D and the development of depression are shown in Table 2 and Supplementary Table 2. The locations of the studies were classified as Europe and Asia. Participants in these studies varied in age from 18 to 79 years old with 878 cases in both the experimental groups and the control groups. Dosage of vitamin D ranged from 1,500 IU per day to about 7,143 IU per day. The follow-up duration varied between 4 and 52 weeks. On the basis of the Cochrane Collaboration tool, particulars concerning the quality assessment of 29 studies are shown in Supplementary Figures 1, 2.

**Table 1:** The characteristics of studies included in this meta-analysis (the correlation between vitamin D and the incidence of depression).

**Author, year**	**Country**	**Follow-up (weeks)**	**Dose of experimental group**	**Sample size**		**Participants inclusion criteria**	**Depression assessment scale**
				**Experiment**	**Control**		
Jalali-Chimeh et al. (24)	Iran	8	300,000 IU/4 weeks	38	38	BMI: 18.5–24.9 kg/m^2^, Age: 18–45 years for women with sexual dysfunction	BDI
Jorde et al. (27)	Norway	48	40,000 IU/week 20,000 IU/week	116 106	112	Obese adults	BDI
Dean et al. (20)	Australia	6	5,000 IU/day	63	65	Age ≥ 18 years for healthy adults	BDI
Kjærgaard et al. (28)	Norway	24	40,000 IU/week	120	110	Age: 30 to 75 years with low 25(OH)D levels	BDI-II, HADS, MADRS
Bertone-Johnson et al. (18)	USA	96	400 IU/day	1109	1143	Age: 50–79 years for postmenopausal women	Burnam scale
Frandsen et al. (21)	Denmark	12	2,800 IU/day	22	21	Healthy adults	SIGH-SAD
Mason et al. (30)	USA	48	2,000 IU/day	109	109	BMI ≥ 25 kg/m^2^, Age: 50–75 years for postmenopausal womenwith low 25(OH)D levels	BSI-18
Vaziri et al. (34)	Iran	12	2,000 IU/day	75	78	Age ≥ 18 years for healthy pregnant women	EPDS
Rolf et al. (33)	the Netherlands	48	14,000 IU/day	20	20	Age: 18–55 years with multiple sclerosis	HADS-D
Grung et al. (23)	Norway	21-28	1,600 IU/day	23	23	Age: 13–14 years for healthy adolescents	YSR-CBCL
Ghaderi et al. (22)	Iran	12	50,000 IU/fortnight	34	34	Age: 25–70 years with methadone treatment	BDI
Jorde and Kubiak (26)	Norway	16	100,000 IU bolus and 20,000 IU/week 100,000 IU bolus and 20,000 IU/week (+psychopharmaca)	192 14	193 9	Age > 40 years with low 25(OH) D levels	BDI-II
Mousa et al. (31)	Australia	16	100,000 IU bolus and 4,000 IU/day	26	22	BMI ≥ 25 kg/m^2^ or BMI≥ 29 kg/m^2^, Age: 18–60 years with low 25(OH) D levels	BDI-II
Raygan et al. (32)	Iran	12	50,000 IU/fortnight	30	30	Age: 45–85 years for diabetics with CHD	BDI
Jamilian et al. (25)	Iran	12	50,000 IU/fortnight	30	30	Age: 18–40 years for women with polycystic ovary syndrom	BDI-II, DASS
Choukri et al. (19)	New Zealand	24	50,000 IU/months	76	74	Age: 18–40 years for healthy adult women	CES-D
Krivoy et al. (29)	Israel	8	14,000 IU/week	24	23	Age: 18–65 years for schizophrenic patients with clozapine treatment and low 25(OH)D levels	CDS
Fazelian et al. (35)	Iran	16	50,000 IU/fortnight	26	25	Age: 20–60 years for T2DM women with low 25(OH) D levels	DASS-21

*BMI, body mass index; BDI, Beck Depression Inventory; HADS, Hospital Anxiety and Depression Scale; MADRS, Montgomery-Asberg Depression Rating Scale; GDS, Geriatric Depression Scale; SIGH-SAD, The Structured Interview Guide for the Hamilton Rating Scale for Depression-Seasonal Affective Disorder version; BSI, Brief Symptom Inventory Depression Subscale; EPDS, Edinburgh Postnatal Depression Scale; YSR-CBCL, Youth Self-report version of the Child Behavior Checklist; DASS, Depression Anxiety and Stress Scales; CES-D, Center for Epidemiological Studies Depression Scale; CDS, Calgary Depression Scale; CHD, coronary heart disease; T2DM, type 2 diabetes; HT, hormone therapy*.

**Table 2:** The characteristics of studies included in this meta-analysis (the correlation between vitamin D and the prognosis of depression).

**Author, year**	**Country**	**Follow-up (weeks)**	**Dose of experimental group**	**Sample size**		**Participants inclusion criteria**	**Depression assessment scale**
				**Experiment**	**Control**		
Yalamanchili and Gallagher (36)	USA	144	200 IU/day (+HT) 200 IU/day	122 123	120 123	Age: 65–77 years for postmenopausal women	GDS-30
Khoraminya et al. (40)	Iran	8	1,500 IU/day	20	20	Age: 18–65 years with MDD and deficiency of vitamin D	BDI-II, HAM-D
Mozaffari-Khosravi et al. (42)	Iran	12	300,000 IU/3 months 150,000 IU/ 3 months	39 36	34	Age: 18–65 years with MDD, Free from other psychiatric diagnoses, any antidepressant drugs or dietary supplements	BDI-II
Wang et al. (44)	China	52	50,000 IU/week	362	364	Age: 20 to 60 years for dialysis patients with MDD and deficiency of vitamin D, Free from other psychiatric diagnoses,any antidepressant drugs or dietary supplements	BDI-II
Sepehrmanesh et al. (43)	Iran	8	50,000 IU/week	18	18	Age: 18–65 years with MDD	BDI-II
Hansen et al. (39)	Denmark	24	2,800 IU/day	28	34	Age: 50–79 years with MDD	HAM-D17, MDI
Alavi et al. (37)	Iran	8	50,000 IU/week	39	39	Age > 60 years with MDD and deficiency of vitamin D, Free from other psychiatric diagnoses	GDS-15
Zhang et al. (45)	China	8	100,000 IU/week	58	65	Age ≥ 18 years for recurrent pulmonary tuberculosis patients with MDD	BDI-II
Amini et al. (38)	Iran	8	50,000 IU/fortnight 50,000 IU/fortnight(+Ca)	26 26	24	BMI ≤ 35 kg/m^2^ for women with PPD	EPDS
Libuda et al. (41)	Germany	4	2,640 IU/day	56	57	Patients with MDD and low 25(OH)D levels	BDI-II, DISYPS-II
Abiri et al. (7)	Iran	8	50,000 IU/week (+Mg) 50,000 IU/week	25 26	26 25	BMI: 35 kg/m^2^, Age > 20 years for women with MDD and low 25(OH)D levels	BDI-II

*MDD, Major depressive disorder; PPD, Postpartum depression; BMI, body mass index; NA, no available; BDI, Beck Depression Inventory; HAM-D, Hamilton Depression Rating Scale; MDI, Major depression inventory; GDS, Geriatric Depression Scale; EPDS, Edinburgh Postnatal Depression Scale; DISYPS-II, Diagnostic System for Mental Disorders in Children and Adolescents according to ICD-10 and DSM-IV*.

### Overall effects and subgroup analysis of vitamin D and the incidence of depression

**Figure 2:**
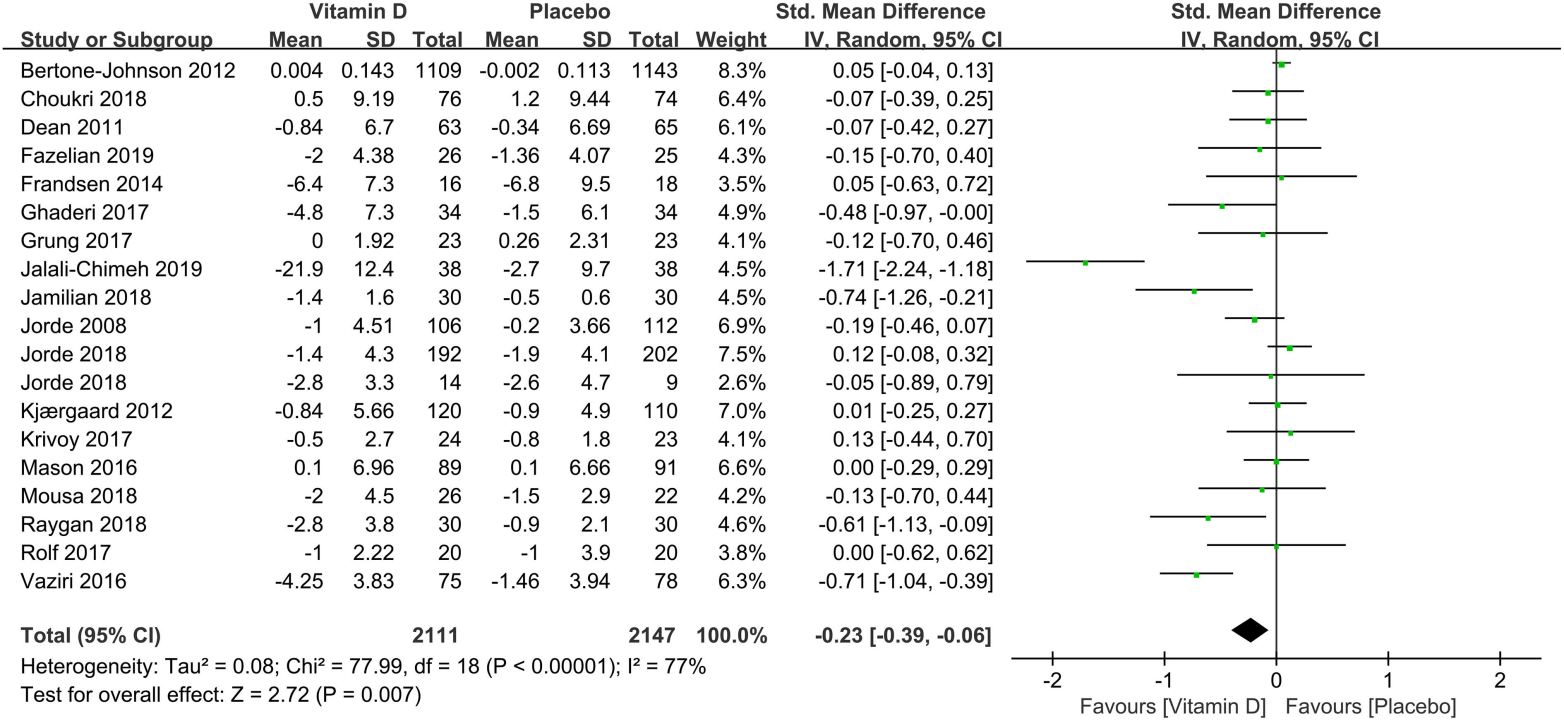
Forest plot of the correlation between vitamin D and the incidence of depression.

A pooled analysis of 18 studies demonstrated the overall effects of the correlation between vitamin D supplementation and the occurrence of depression. Despite the high heterogeneity, the depression assessment scale scores from baseline to terminal in the experimental groups have decreased compared with those in the control groups (SMD: −0.23) (Figure 2), which revealed that the usage of vitamin D supplements might reduce the incidence of depression.

**Figure 3:**
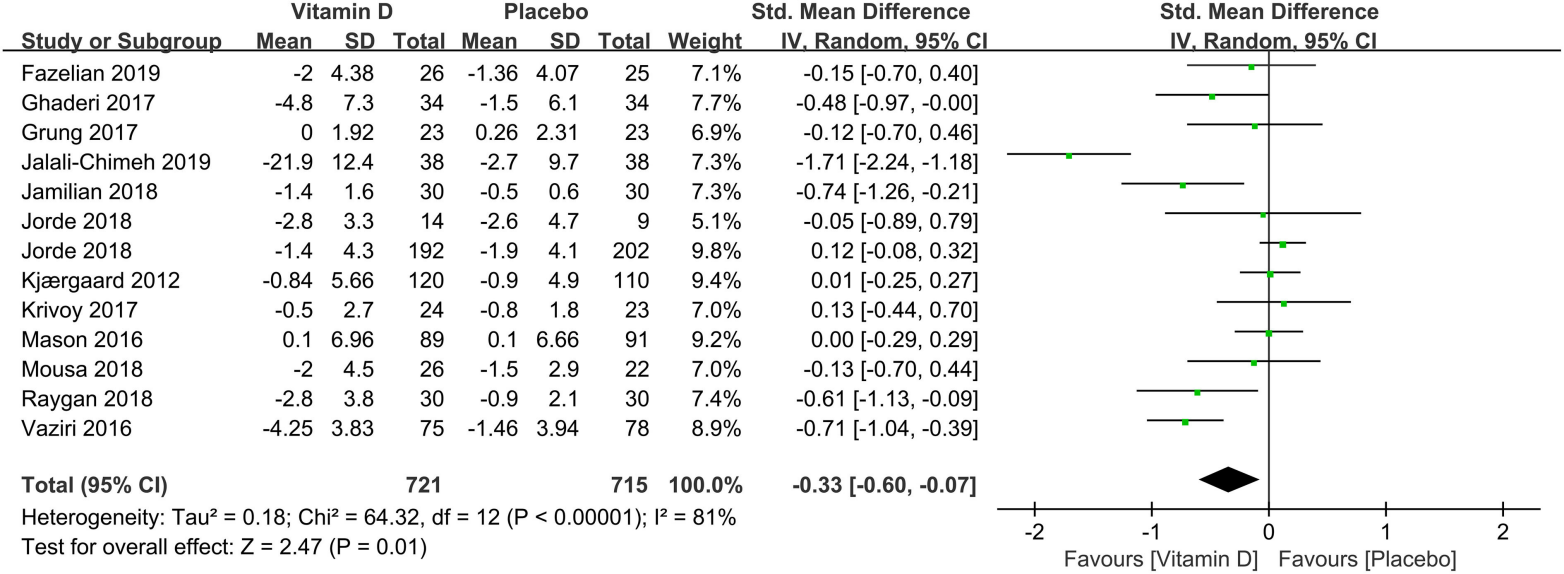
Forest plot of the correlation between vitamin D and the incidence of depression for population with low vitamin D levels (<50 nmol/L).

**Figure 4:**
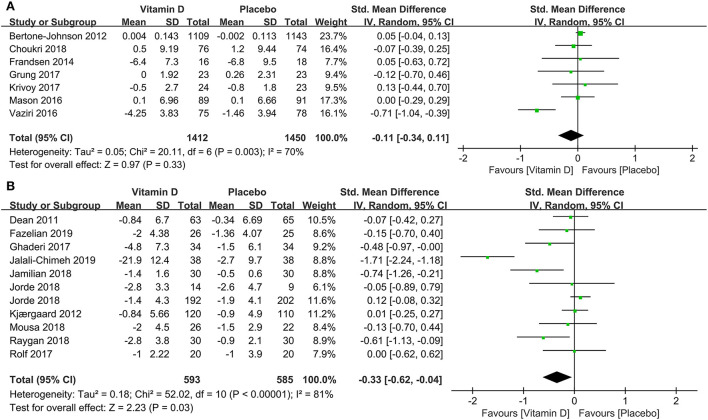
Forest plot of the correlation between supplementary doses of vitamin D and the incidence of depression. **(A)** low doses (≤2,800 IU/day); **(B)** high doses (>2,800 IU/day).

In subgroup analysis, the correlation between vitamin D supplementation and the occurrence of depression based on 1,436 participants with low levels of serum 25(OH)D at baseline were conducted in 12 studies. Compared to the control group, the experimental group had a significant effect on reducing the incidence of depression [SMD: −0.33; 95%CI: (−0.60, −0.07); *p* = 0.01] (Figure 3). To compare the effects of low doses (≤2,800 IU/day) and high doses (>2,800 IU/day) of vitamin D supplementation, seven and 10 studies were examined, respectively. The pooled effects (SMD) for low doses and high doses of vitamin D supplements were −0.11 (Figure 4A) and −0.33 (Figure 4B), respectively. The results revealed that vitamin D supplementation in high doses might promote a decrease in the incidence of depression, while low doses of vitamin D supplements were futile.

In nine studies where the BDI or BDI-II was used as the depression assessment scale, the use of vitamin D might also be helpful to the reduction of the incidence of depression compared with the control group (SMD: −0.36). Vitamin D supplements were shown in 13 RCTs to have effectively decreased the incidence of depression among people with normal weight (SMD: −0.28). On the contrary, it was useless for the overweight people in 4 RCTs (SMD: −0.11). Also, seven studies reported a significant diminution compared to the control group in the incidence of depression in females among 2,922 participants with the vitamin D supplementation (SMD: −0.44). Nevertheless, no apparent change relating to vitamin D supplementation was discovered neither in the elderly (SMD: 0.04) through two studies nor in the older female (SMD: 0.04) through other two studies. Finally, among those who received vitamin D supplements, there was no significant difference between the two groups for the intervention duration ≤8 weeks (SMD: −0.55). However, it was worth noting that when the intervention duration is ≥8 weeks, vitamin D supplementation might be conducive to the decrease in the occurrence of depression compared with the control group (SMD: −0.15) (Table 3).

**Table 3:** Subgroup analysis of the correlation between vitamin D and the incidence/prognosis of depression.

**Subgroup**		**No. of studies**	**Participants**	**Changes in depression**	**SMD**	**95%CI**	***p***	**Heterogeneity(I^2^) (%)**
Occurrence
BDI		9	1,305	↓	−0.36	−0.65–(−0.07)	0.02	83
Overweight (BMI >24 kg/m^2^)		4	497	-	−0.11	−0.29–0.06	0.21	0
Nomal weight (BMI: 18–24kg/m^2^)		13	3,721	↓	−0.28	−0.49–(−0.07)	0.01	83
The elderly (≥50 years old)		2	2,432	-	0.04	−0.04–0.12	0.29	0
Female		7	2,922	↓	−0.44	−0.81–(−0.06)	0.02	91
Older female		2	2,432	-	0.04	−0.04–0.12	0.29	0
Intervention duration	≤ 8 weeks	3	251	-	−0.55	−1.61–0.52	0.32	93
	≥8 weeks	16	4,054	↓	−0.15	−0.28–(−0.01)	0.03	60
Development
BDI		7	1,236	-	−1.04	−2.29–0.21	0.10	99
Overweight (BMI >24 kg/m^2^)		2	178	↓	−0.79	−1.34–(−0.24)	0.005	72
Nomal weight (BMI: 18–24 kg/m^2^)		4	445	↓	−0.34	−0.68–0.00	0.05	60
Female		3	448	↓	−0.61	−1.16–(−0.06)	0.03	84
Intervention duration	≤ 8 weeks	7	555	↓	−0.69	−1.08–(−0.30)	<0.001	80
	≥8 weeks	10	1,656	↓	−1.00	−1.90–(−0.09)	0.03	98

*SMD, standard mean difference; BMI, body mass index; BDI, Beck Depression Inventory*.

*-, There was no statistical difference in the change of depression scores between the experimental and control groups*.

*↓, The depression scores of the experimental group decreased compared with control groups*.

### Overall effects and subgroup analysis of vitamin D and the prognosis of depression

**Figure 5:**
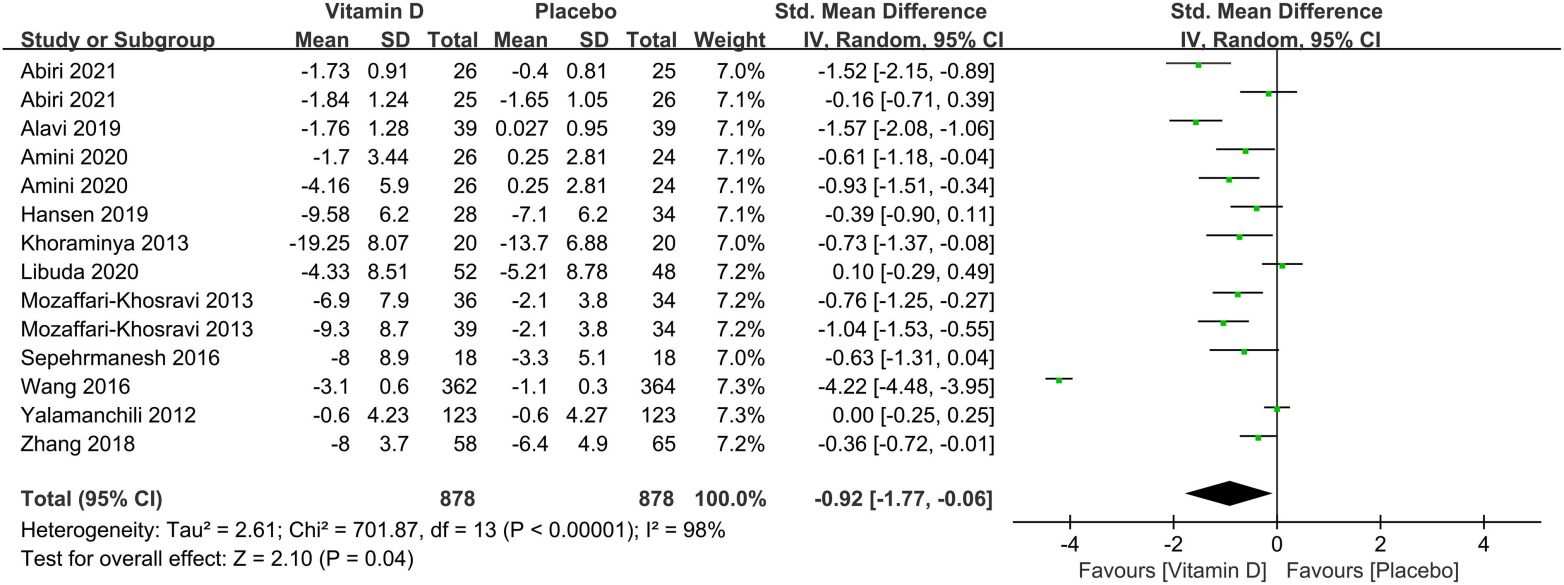
Forest plot of the correlation between vitamin D and the prognosis of depression.

To probe the overall effects of the correlation between vitamin D supplementation and the prognosis of depression, 11 studies were included in a pooled analysis. From baseline to terminal, although there was also high heterogeneity, depression assessment scale scores showed a significant decline (SMD: −0.92) (Figure 5), indicating that the interventions with vitamin D supplements might be beneficial to the treatment of depression.

**Figure 6:**
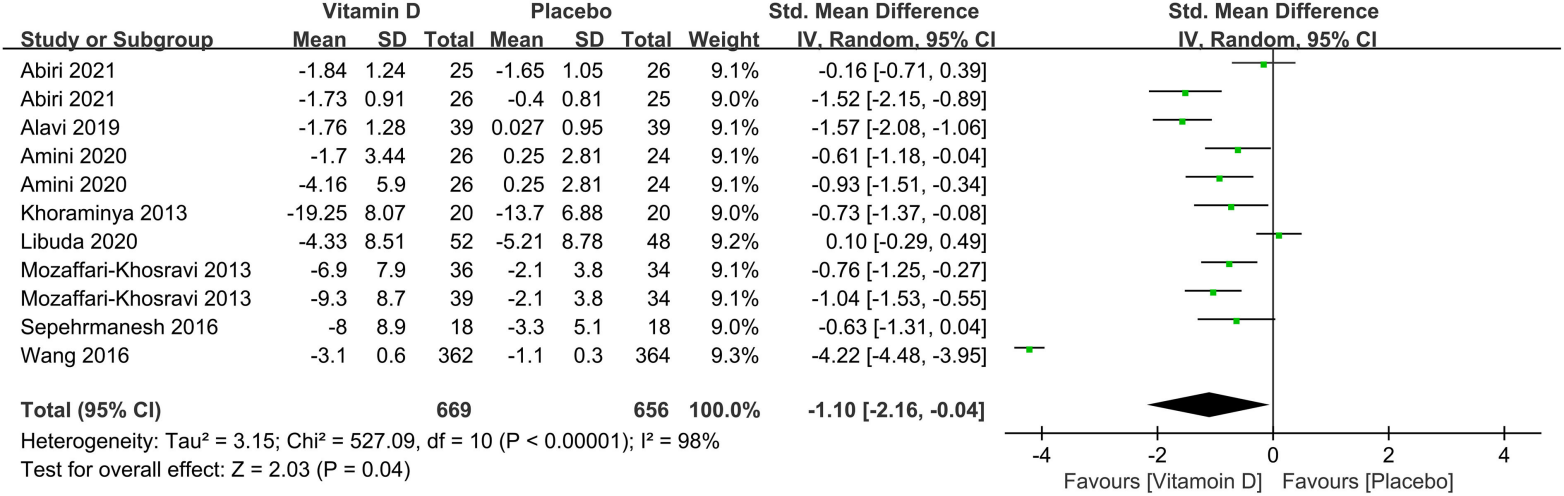
Forest plot of the correlation between vitamin D and the prognosis of depression for patients with low vitamin D levels (<50 nmol/L).

**Figure 7:**
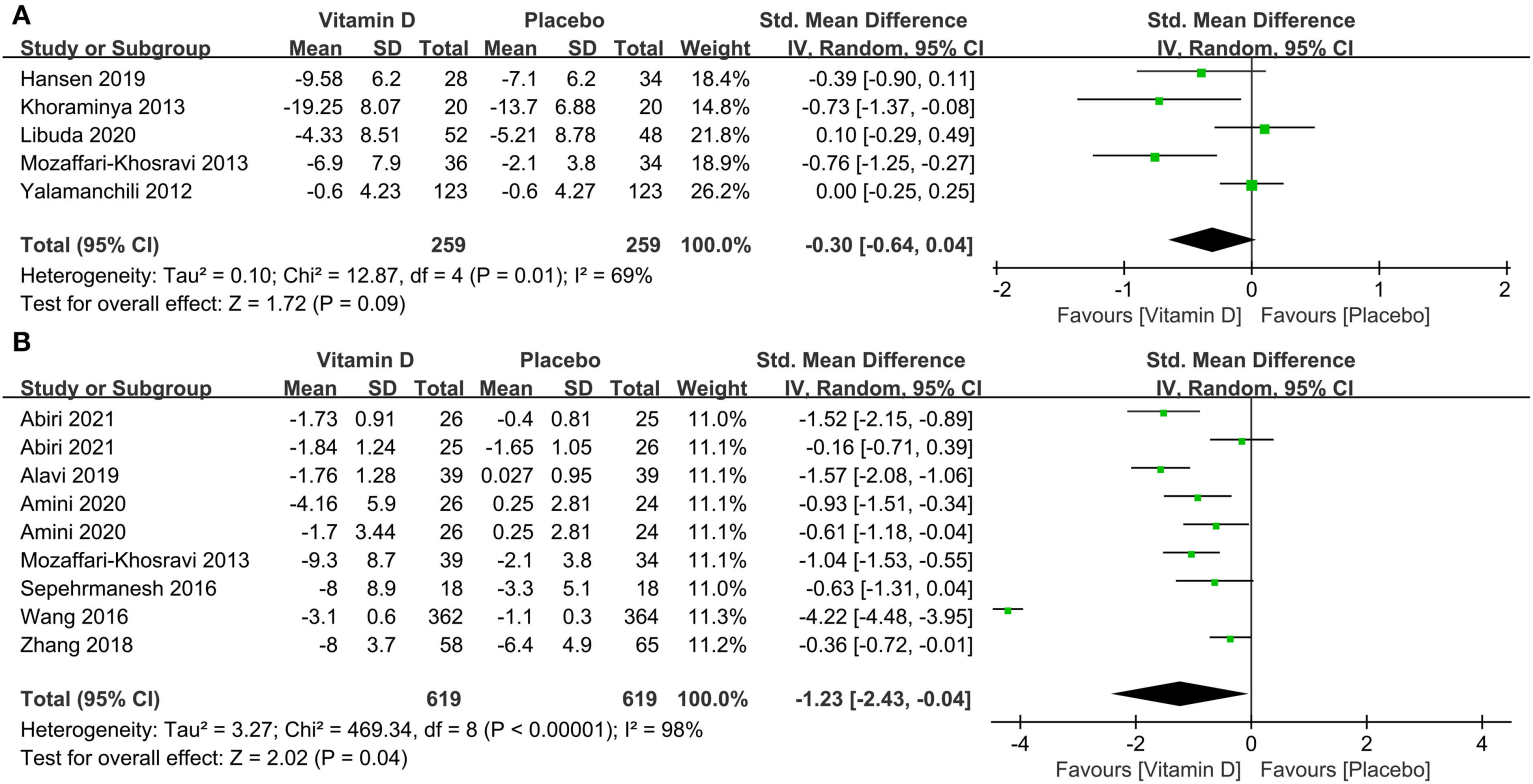
Forest plot of the correlation between supplementary doses of vitamin D and the prognosis of depression. **(A)** low doses (≤2,800 IU/day); **(B)** high doses (>2,800 IU/day).

In subgroup analysis, eight studies compared the effects among 1,325 participants with a low level of serum 25(OH)D at baseline between the two groups. The results illustrated that compared to the control group, those with depression and low serum vitamin D levels might benefit from vitamin D supplementation (SMD: −1.10) (Figure 6). The correlation between vitamin D usage and the development of depression among low doses (≤2,800 IU/day) and high doses (>2,800 IU/day) of vitamin D supplementation were examined in five and seven studies, separately. The results demonstrated that high-dose vitamin D supplementation might be more favorable in the depression therapy, whereas low-dose vitamin D supplementation was relatively ineffective by comparing parameters with the control group, the pooled effects (SMD) for low-dose analysis was −0.30 (Figure 7A) and high-dose supplementation was −1.23 (Figure 7B), respectively.

However, in the subset of seven studies where the BDI or BDI-II was used as the depression assessment scale, no significant difference was found (SMD: −1.04). With additional consideration of patients' BMI, vitamin D supplementation was effective in the treatment of depression both in people with normal weight (SMD: −0.34) in four studies and those who were overweight (SMD: −0.79) in another two studies. In three RCTs where the vitamin D was supplied to 448 females, compared to the control group, a significant therapeutic effect on depression was also observed (SMD: −0.61). Last but not least, vitamin D supplementation was beneficial as an adjunct to the treatment of depression, compared with the control group, not only for the intervention duration ≥8 weeks (SMD: −1.00) but also for the intervention duration ≤8 weeks (SMD: −0.69; 95%CI: [−1.08, −0.30]; *p* < 0.001) (Table 3).

### Publication bias and sensitivity analysis

To evaluate publication bias, the funnel plots of the publication bias of vitamin D and the incidence/prognosis of depression was derived (Supplementary Figures 3, 4). All the funnel plots in our results seem to be symmetrical with the effect estimates, which indicated that there was no significant publication bias in our studies. Sensitivity analysis was also conducted to estimate the stability of the pooled effects by excluding each study successively, and the results were supportive to prove the stability of our results.

## Discussion

In the present systematic review, we summarized the published outcomes related to the effect of vitamin D supplements on depression. A meta-analysis based on 18 studies was conducted to evaluate the correlation between vitamin D supplementation and the incidence of depression whereas the correlation between vitamin D supplementation and the prognosis of depression from 11 studies was estimated. Congruent with the previous systematic reviews and meta-analysis (13, 46, 47), in our main study, vitamin D supplementation was found to be associated with a decrease in the occurrence of depression as well as an improvement in the treatment of depression. The impact was also observed in subgroup analyses of serum 25(OH)D levels, doses of vitamin D supplementation, BMI, gender, and intervention duration. Consequently, our results may be supportive to consider vitamin D supplementation to be effective in lowering the risk and improving the treatment of depression.

So far, the correlation between vitamin D and mental health has been investigated mostly in the context of depression or depressive symptoms (2, 12, 46–50), with several putative mechanisms for the effect of vitamin D being proposed (51). Vitamin D has been discovered to have effects on the central nervous system. Some studies have shown that vitamin D receptors are widely distributed in areas of the brain, such as the amygdala and substantia nigra (52). Vitamin D is able to cross the blood-brain barrier, activate vitamin D receptors, and play a role in human behavior control (53).

In addition, in recent years, the cyclo-AMP responsive element-binding protein (CREB) cascade and its effect on the brain-derived neurotrophic factor (BDNF) have made some rationales for the pathogenesis and treatment of depression (54). The hypothesis of catecholamine and serotonin deficiency suggests that depression may be largely caused by the lack of monoamines (norepinephrine and serotonin) in the synaptic space (55). Vitamin D, however, weighs on the synthesis of monoamines and may regulate the activity of GABA-A receptors to some extent (52, 56, 57). By regulating the release of neurotransmitters and the synthesis of neurotrophic factors, vitamin D is relevant in ameliorating mood and behavior in humans (58). Meanwhile, the function of vitamin D may be attributed to its neuroprotective effect on the brain, as it has been proved that vitamin D is able to lower plasma C-reactive protein in patients with psychiatric disorders and modulate inflammation by suppressing proinflammatory cytokines (59, 60).

Although the relationship between vitamin D and depression has been extensively discussed, the evidence in terms of data and mechanism appears to be logical. Several studies (12, 49) have found that taking vitamin D supplements does not result in a substantial reduction in depression. These might stem from the biological defects of the preliminary studies, two studies (13, 50) revealed that those unflawed trials contributed to a statistically prominent improvement in depression. Similarly, a significant association between the use of vitamin D and depression by Anglin agreed with the previous finding (2). Moreover, in the meta-analysis by Shaffer (46), vitamin D was shown to be beneficial in relieving depressive symptoms in patients with clinically significant depression as well.

As for the assessment scale that was used to measure symptoms of depression, in this study, when taking into account the most frequently used assessment scale BDI and BDI-II only, vitamin D supplementation was only effective in the prevention of depression, not in the treatment of depression. This issue might be owing to the fact that the level of depression was incapable of being estimated with meta-regression when under different measurements (13). It served as a reminder to pay attention to the impact of various measurement tools.

In line with prior meta-analyses that have been reported (2, 61), in terms of the impact of the serum vitamin D level on depression, the subgroup analysis showed that patients with low vitamin D levels (<50 nmol/L) benefit from supplementation in both prevention and treatment of depression. Vitamin D deficiency is defined as serum 25(OH)D levels of <50 nmol/L, with levels of 50–75 nmol/L being deemed insufficiency (62). The prevalence of neurodegenerative, neuroinflammatory, and neuropsychological disorders has been linked to hypo-vitamin D in studies based on humans. In a large cohort study (63), higher serum vitamin D levels were authenticated to be associated with a lower incidence of depression at follow-up, suggesting that vitamin D deficiency may signify a latent biological vulnerability for depressive disorder. Hence, it comes as no surprise that depressive patients with hypovitaminosis D are more prone to be relieved from adequate vitamin D supplementation. Furthermore, patients with hypovitaminosis D are supposed to be more alert to the incidence of depression to some extent. It is advised that individuals with vitamin D deficiency should take suitable vitamin D supplements, including dietary sources or pills.

Concerning the BMI, our results revealed that supplementing with vitamin D worked well in both prevention and treatment in people with normal weight, whereas only treatment and not prevention in those who were overweight. According to previous studies (27, 64), BMI appeared to have an essential mediating role in the relationship between vitamin D and depression, and vitamin D supplements might improve depressive symptoms in obese patients. As mentioned before, in the overweight population, no significant difference was observed in the prevention group. The different findings might be ascribed to the truth that only a small proportion of the studies in subgroup analysis included assessed the effect of vitamin D supplementation on overweight patients in the aspect of prevention. Moreover, another contributing factor might contain differences in the study population, variable vitamin D supplementation dosages, and differences in baseline serum 25 (OH) D concentrations levels. A recent comparative observational study showed that the decrease in serum 25 (OH) D level in obese adults was related to incident depression, while the proportion of having vitamin D deficiency in depressed people was higher than that in non-depressed people (65). Vitamin D deficiency was also proved to be less common in healthy individuals (66). Due to the large body and adipose mass, overweight people might have a low increase in serum vitamin D level and require more vitamin D than normal (67), a regular dose of vitamin D would possibly be insufficient for them. Besides, the adipose mass could also lead to higher systemic inflammation, so higher doses of vitamin supplements were also considered necessary. As a result, the regular dose of vitamin D supplementation might not be as effective in overweight people without a depressive disorder.

Intriguingly, the subgroup analysis of gender demonstrated that vitamin D supplements significantly alleviated depression scores in all females, regardless of whether they were depressed or not. A previous study (68) found that female patients with depressive symptoms improved significantly more following vitamin D administration than male patients, which was in accordance with our findings. The mechanism might involve that vitamin D has a bearing on the synthesis of serotonin and a moderate amount of vitamin D may elevate the level of extracellular serotonin (69), particularly in females, so as to improve the symptoms of depressive disorder. Concerning vitamin D supplementation was ineffective among the elderly population in our results, due to the small number of studies and the unavailability of research data, the outcomes were tough to explain. Additionally, according to a pilot study (70), even though there was a correlation between an increase in vitamin D levels and a decrease in depressive symptom scores, the association vanished as the research population over 65 years old. Furthermore, since most included studies defined depression as being above the dividing point of the depression scale with no adjustment for physical frailty, the outcome could be easily influenced by the confounding factors as well and should be treated with more caution.

When it comes to the strategy of vitamin D supplement, considering the supplementary dosage, the replenishment threshold was set at 2,800 IU each day. Our findings illustrated that high doses (>2,800 IU/day) were beneficial in the prevention and treatment of depression, while low doses (≤2,800 IU/day) were regarded as ineffective. The common dose of vitamin D supplements for adults is 800 IU per day, with a range from 400 to 2,000 IU per day depending on age, weight, illness status, and race (71). Besides, 50,000 IU is acknowledged to be the upper limit of the recommended daily intake (72–75), excessive intake might result in toxicity and some adverse reactions such as hypercalciuria (76). Yet owing to the limited number of studies included in the treatment group in terms of low-dose supplementation, the relevant results should be interpreted with caution either. As for the intervention duration, “8 weeks” was recognized as the time point that might trigger the response to vitamin D, whether in the prevention or treatment groups. Although a previous study showed that the response to vitamin D could not be observed even at 20 weeks, this was based on a status of relative vitamin D repletion (77). As a secosteroid hormone, vitamin D functions through transcription in the nucleus, which takes a long time to work. This might be a plausible explanation for why observing the response to vitamin D took so long. Unexpectedly, we also observed a vitamin D response in the treatment group under the circumstances that the intervention duration was <8 weeks. This phenomenon can also be interpreted by the fact that the mean level of vitamin D in depressed people is more likely to be lower than that in non-depressed people, making depressed ones more sensitive to vitamin D administration. Anyway, our analysis indicated the effects of vitamin D with daily supplementary doses >2,800 IU and an intervention duration of 8 weeks was significant. High heterogeneity was also observed in some subgroups in our meta-analysis, which could be attributed to several reasons. Initially, due to the differences in race, outdoor activity intensity, sunshine time, diet, etc., the baseline and terminal serum vitamin D levels between participants also exist lot of discrepancies. Besides, on account of the differences in the cognition of mental diseases among different populations, there was also a discrepancy in the results of the depression assessment scale to some extent.

### Strengths and limitations

To our knowledge, this is the first comprehensive meta-analysis to assess the correlation between vitamin D and the incidence as well as the prognosis of depression. And there were several strengths to our study. First, more recent RCTs were incorporated into this study, thereby updating previous findings. Additionally, although the tool evaluation constructs that were used to measure the psychological symptoms of subjects varied slightly, compared with previous studies (13, 59), they were limited within the depression assessment scale, which decreased the impact of other emotional or physical symptoms on confounding factors of outcomes.

Nonetheless, some associated limitations of our study should be mentioned. First, we have not pre-registered a protocol for this meta-analysis, which might introduce potential bias to the review. Second, due to the race, the intensity of outdoor activity, sun exposure time, dietary differences, and cognition of mental diseases in different ethnic groups and other factors, the high heterogeneity in some subgroups of our meta-analysis was predictable. Third, detailed information such as simultaneous psychosocial interventions, use of calcium supplementation, and antidepressants were not provided in a few studies, our results might be disturbed by overlapping factors. As a consequence, the possibility that the observed amelioration of psychological state in respondents might be the result of overlapping elements acting together should be taken into account. Fourth, the relationship between serum vitamin D levels and the severity of depression was not examined in this study. Also, vitamin D supplementation cannot be fully equivalent to an increase in serum vitamin D levels. Hence, relevant findings should be treated with more care.

## Conclusion

To conclude, this meta-analysis demonstrates that vitamin D has a positive impact on both the decreased incidence of depression and a better prognosis of depression. The impact is consistently observed in subgroup analyses of serum 25(OH)D levels, doses of vitamin D supplementation, BMI, gender, and intervention duration. Whether suffering from depression or not, individuals with low vitamin D levels and females are most likely to benefit from vitamin D supplementation. A daily supplementary dose of 2,800 IU and an intervention duration of 8 weeks are considered as the point that may cause the observational effect of vitamin D. Besides, our results also reveal that vitamin D supplementation works well in both prevention and treatment in normal-weight people, whereas only in treatment not prevention for those who are overweight.

## Author contributions

LR designed the research process. FX and TH searched the database for corresponding articles and drafted the meta-analysis. DL extracted useful information from the articles above. RF used statistical software for analysis. CN and JH polished this article. All authors contributed to manuscript revision, read, and approved the submitted version.

## Conflict of interest

The authors declare that the research was conducted in the absence of any commercial or financial relationships that could be construed as a potential conflict of interest.

## Publisher's note

All claims expressed in this article are solely those of the authors and do not necessarily represent those of their affiliated organizations, or those of the publisher, the editors and the reviewers. Any product that may be evaluated in this article, or claim that may be made by its manufacturer, is not guaranteed or endorsed by the publisher.

## Supplementary material

Supplementary Table 1 The characteristics of studies included in this meta-analysis (the correlation between vitamin D and the incidence of depression).

Supplementary Table 2 The characteristics of studies included in this meta-analysis (the correlation between vitamin D and the prognosis of depression).

Supplementary Figure 1 Quality assessment of studies included in this meta-analysis (risk of bias graph).

Supplementary Figure 2 Quality assessment of studies included in this meta-analysis (Risk of bias summary).

Supplementary Figure 3 Funnel plot for publication bias (the correlation between vitamin D and the incidence of depression).

Supplementary Figure 4 Funnel plot for publication bias (the correlation between vitamin D and the prognosis of depression).
