# Supplementary material for: Effect of vitamin D supplementation on the incidence and prognosis of depression: An updated meta-analysis based on randomized controlled trials
Source: Front Public Health. 2022 Aug 1;10:903547. doi: 10.3389/fpubh.2022.903547 (PMC9376678; doi:10.3389/fpubh.2022.903547)
Supplement: Supplementary file 1 [file Data_Sheet_1.pdf]

Search strategies:

Search Details: ((("vitamin d"[MeSH Terms] OR "vitamin d"[All Fields] OR "ergocalciferols"[MeSH Terms] OR "ergocalciferols"[All Fields] OR (("vitamin d"[MeSH Terms] OR "vitamin d"[All Fields] OR "ergocalciferols"[MeSH Terms] OR "ergocalciferols"[All Fields]) AND ("supplemental"[All Fields] OR "supplementating"[All Fields] OR "supplementation"[All Fields] OR "supplementation s"[All Fields] OR "supplementations"[All Fields] OR "supplemention"[All Fields])) OR ("25 hydroxyvitamin d"[Supplementary Concept] OR "25 hydroxyvitamin d"[All Fields] OR "25 hydroxyvitamin d"[All Fields] OR "calcifediol"[MeSH Terms] OR "calcifediol"[All Fields]) OR (("25 hydroxyvitamin d"[Supplementary Concept] OR "25 hydroxyvitamin d"[All Fields] OR "25 hydroxyvitamin d"[All Fields] OR "calcifediol"[MeSH Terms] OR "calcifediol"[All Fields]) AND ("supplemental"[All Fields] OR "supplementating"[All Fields] OR "supplementation"[All Fields] OR "supplementation s"[All Fields] OR "supplementations"[All Fields] OR "supplemention"[All Fields])) OR ((25[UID] AND ("hydroxide ion"[Supplementary Concept] OR "hydroxide ion"[All Fields] OR "oh"[All Fields])) AND "D"[All Fields]) OR ("cholecalciferol"[MeSH Terms] OR "cholecalciferol"[All Fields] OR "cholecalciferols"[All Fields] OR "colecalciferol"[All Fields])) AND ("depressed"[All Fields] OR "depression"[MeSH Terms] OR "depression"[All Fields] OR "depressions"[All Fields] OR "depression s"[All Fields] OR "depressive disorder"[MeSH Terms] OR ("depressive"[All Fields] AND "disorder"[All Fields]) OR "depressive disorder"[All Fields] OR "depressivity"[All Fields] OR "depressive"[All Fields] OR "depressively"[All Fields] OR "depressiveness"[All Fields] OR "depressives"[All Fields])) OR ("depressed"[All Fields] OR "depression"[MeSH Terms] OR "depression"[All Fields] OR "depressions"[All Fields] OR "depression s"[All Fields] OR "depressive disorder"[MeSH Terms] OR ("depressive"[All Fields] AND "disorder"[All Fields]) OR "depressive disorder"[All Fields] OR "depressivity"[All Fields] OR "depressive"[All Fields] OR "depressively"[All Fields] OR "depressiveness"[All Fields] OR "depressives"[All Fields]) OR (("negative"[All Fields] OR "negatively"[All Fields] OR "negatives"[All Fields] OR "negativities"[All Fields] OR "negativity"[All Fields]) AND ("emoting"[All Fields] OR "emotion s"[All Fields] OR "emotions"[MeSH Terms] OR "emotions"[All Fields] OR "emotion"[All Fields] OR "emotional"[All Fields] OR "emotive"[All Fields])) OR ("depressive disorder, major"[MeSH Terms] OR ("depressive"[All Fields] AND "disorder"[All Fields] AND "major"[All Fields]) OR "major depressive disorder"[All Fields] OR ("major"[All Fields] AND "depressive"[All Fields] AND "disorder"[All Fields]) OR "major depressive disorder"[All Fields] OR "depressive disorder"[MeSH Terms] OR ("depressive"[All Fields] AND "disorder"[All Fields]) OR "depressive disorder"[All Fields] OR ("major"[All Fields] AND "depressive"[All Fields] AND "disorder"[All Fields]))) AND ("placeboes"[All Fields] OR "placebos"[MeSH Terms] OR "placebos"[All Fields] OR "placebo"[All Fields])

https://pubmed.ncbi.nlm.nih.gov/?term=%28%28%28%28%28%28%28%28%28%28vitamin+D%29+OR+%28vitamin+D+supplementation%29%29+OR+%2825-hydroxyvitamin+D%29%29+OR+%2825-hydroxyvitamin+D+supplementation%29%29+OR+%2825%28OH%29D%29%29+OR+%28cholecalciferol%29%29+AND+%28depression%29%29+OR+%28depressive%29%29+OR+%28negative+emotion%29%29+OR+%28major+depressive+disorder%29%29+AND+%28placebo%29&sort=
