# Supplementary material for: Effect of vitamin D supplementation on the incidence and prognosis of depression: An updated meta-analysis based on randomized controlled trials
Source: Front Public Health. 2022 Aug 1;10:903547. doi: 10.3389/fpubh.2022.903547 (PMC9376678; doi:10.3389/fpubh.2022.903547)
Supplement: Supplementary Table 1 — The characteristics of studies included in this meta-analysis (the correlation between vitamin D and the incidence of depression). [file Table_1.pdf]

Supplementary Table 1. The characteristics of studies included in this meta-analysis (the correlation between vitamin D and the incidence of depression).

Author, year	Trial registration number	Mean age, year		BMI (kg/m^2)		Baseline 25 (OH) D (nmol/L)		Terminal 25 (OH) D (nmol/L)	
		Experiment	Control	Experiment	Control	Experiment	Control	Experiment	Control
Jalali-Chimeh,2019	IRCT201608053860N28	34.9	35.9	NA	NA	38.7 (3.6)	36.9 (4.1)	NA	NA
Jorde, 2008	NCT00243256	47.0; 50.0	53.0	33.5 (28.8–45.0); 33.3 (28.7–46.1)	34.8 (28.6–47.1)	55.2 (16.8–97.0); 52.2 (15.4–111.5)	52.4 (18.5–99.4)	112.1 (46.7–193.4); 87.8 (51.5–162.3)	50.0 (20.3–99.8)
Dean, 2011	ACTRN12610000318088	21.45	22.06	NA	NA	76.25 (19.63)	77.23 (20.95)	98.0 (3.3)	77.2 (2.6)
Kjærgaard, 2012	NCT00960232	53.4	53.3	27.5 (4.0)	28.0 (4.2)	47.4 (15.8)	47.7 (15.5)	147.7 (29.2)	52.5 (16.1)
Bertone-Johnson, 2012	NCT00000611	NA	NA	NA	NA	52.0 (21.1)	52.0 (21.1)	NA	NA
Frandsen, 2014	NCT01462058	44.2	44.4	NA	NA	NA	NA	NA	46.2 (21.7)
Mason, 2016	NCT01240213	59.6	59.6	32.4 (5.8)	32.4 (5.8)	53.5 (12.7)	53.5 (12.7)	NA	NA
Vaziri, 2016	IRCT2015020310327N11	26.40	26.22	NA	NA	32.1 (19.77)	29.73 (16.0)	43.65 (25.23)	30.17 (14.95)
Rolf, 2017	NCT01285401	38.5	37.6	NA	NA	58.0 (38.0-82.0)	53.0 (43.0-63.0)	226.0 (159.0-250.0)	61.0 (44.0-84.0)
Grung, 2017	REK-West2013/1311	NA	NA	NA	NA	44.0 (14.0)	39.0 (16.0)	62.0 (20.0)	44.0 (15.0)
Ghaderi, 2017	IRCT201701035623N102	40.1	42.5	24.6 (4.4)	25.5 (4.4)	34.7 (11.2)	33.7 (11.2)	55.0 (18.7)	32.7 (14.7)
Jorde, 2018	NCT02750293	51.3; 53.0	52.6; 53.0	27.7 (5.0); 29.0 (5.3)	27.9 (4.7); 26.4 (3.5)	32.8 (11.2); 28.3 (9.0)	35.4 (13.7); 29.8 (11.0)	89.3 (18.8); 82.2 (27.3)	30.8 (9.8); 28.1 (8.0)
Mousa, 2018	NCT02112721	31.5	32.0	31.7 (4.9)	30.5 (4.0)	31.7 (13.0)	35.0 (9.5)	87.7 (20.7)	37.9 (4.2)
Raygan, 2018	IRCT2017073033941N4	71.5	67.3	29.0 (6.2)	28.2 (4.9)	36.7 (11.7)	34.4 (7.5)	66.3 (15.3)	34.7 (8.5)
Jamilian, 2018	IRCT201704245623N114	26.8	25.1	27.4 (3.9)	27.1 (7.0)	30.2 (7.8)	31.7 (6.7)	54.7 (15.7)	31.7 (8.0)
Choukri, 2018	ACTRN12613000540718	24.4	23.9	25.1 (4.5)	24.8 (4.9)	77.7 (26.1)	74 (26.1)	84.5 (22.7)	49.2 (28.1)
Krivoy, 2017	NCT01759485	39.4	42.5	28.3 (3.8)	28.1 (6.0)	37.2 (5.9)	42.7 (15.5)	68.6	42.3

Fazelian, 2019	IRCT201709270 36451N1	48.5	46.32	30.21 (4.42)	29.19 (6.41)	55.38 (15.19)	53.84 (16.81)	75.61 (30.39)	58.18 (29.51)

NA, no available; BMI, body mass index.
