# Supplementary material for: Effect of vitamin D supplementation on the incidence and prognosis of depression: An updated meta-analysis based on randomized controlled trials
Source: Front Public Health. 2022 Aug 1;10:903547. doi: 10.3389/fpubh.2022.903547 (PMC9376678; doi:10.3389/fpubh.2022.903547)
Supplement: Supplementary Table 2 — The characteristics of studies included in this meta-analysis (the correlation between vitamin D and the prognosis of depression). [file Table_2.pdf]

Supplementary Table 2. The characteristics of studies included in this meta-analysis (the correlation between vitamin D and the prognosis of depression).

Author, year	Trial registration number	Mean age, year		BMI (kg/m^2)		Baseline 25 (OH) D (nmol/L)		Terminal 25 (OH) D (nmol/L)	
		Experiment	Control	Experiment	Control	Experiment	Control	Experiment	Control
Yalamanchili, 2012	NA	71.4;<br>71.8	71.4;<br>71.1	26.7 (4.3);<br>26.7 (5.4)	27.22 (5.0)	80.0 (29.0);<br>75.75 (23.5)	77.25 (24.75);<br>79.25 (27.5)	NA	NA
Khoraminya,2013	IRCT201201072394N6	38.1	39.65	28.32 (5.43)	29.00 (6.82)	58.8 (10.1)	57.5 (11.0)	117.1(35.4)	NA
Mozaffari-Khosravi, 2013	NA	32.1;<br>32.7	33.0	26.2 (4.8);<br>26.3 (5.1);	25.9 (3.8)	21.3;<br>23.0	25.4	60.2;<br>54.6	28.2
Wang, 2016	2014058	NA	NA	23.7(10.6)	24.2(9.8)	54.8 (10.2)	58 (14.5)	98.8(34.1)	55.3(18.7)
Sepehrmanesh, 2016	IRCT201412065623N29	36.5	36.1	25.8(5.2)	26.9(3.8)	34 (19.7)	23.0 (15.0)	85(22.7)	20.7(10)
Hansen, 2019	NCT01390662	39.6	38.7	NA	NA	43.2 (24.6)	44.3 (24.1)	44.3 (24.1)	52.0 (33.5)
Alavi, 2019	IRCT201604252758N1	68.7	67.0	NA	NA	58.6 (16.1)	55.1 (15.1)	112.9 (24.7)	67.3 (39.7)
Zhang, 2018	20150126	38.3	40.2	21.2 (3.8)	20.7 (4.1)	57.3 (17.7)	61.3 (11.5)	67.7 (20.7)	59.0 (20.3)
Amini, 2020	IRCT2016091416123N9	29.25;<br>26.88	28.92	26.62 (0.84);<br>27.59 (0.54)	27.69 (0.86)	39.83 (31.94,47.69);<br>36.56 (28.67,46.72)	36.74 (28.40,45.05)	58.03 (45.17,70.91);<br>51.39 (39.73,63.04)	42.90 (33.77,52.04)
Libuda, 2020	DRKS00009758	16.1	15.8	23.8 (5.9)	22.8 (5.5)	22.49 (5.12)	22.23 (5.32)	62.1 (15.87)	27.0 (11.19)
Abiri, 2021	IRCT20090822002365N23	34.40;<br>34.15	34.30;<br>34.36	33.00 (1.93);<br>33.47 (1.82)	33.18 (1.92);<br>33.40 (1.93)	42.47 (22.49);<br>42.81 (21.43)	42.81 (22.10);<br>42.41 (25.12)	99.48 (18.39);<br>98.75 (15.53)	38.18 (20.78);<br>38.13 (20.65)

NA, no available; BMI, body mass index.
