# Supplementary figures and images for: Effect of vitamin D supplementation on the incidence and prognosis of depression: An updated meta-analysis based on randomized controlled trials
Source: Front Public Health. 2022 Aug 1;10:903547. doi: 10.3389/fpubh.2022.903547 (PMC9376678; doi:10.3389/fpubh.2022.903547)

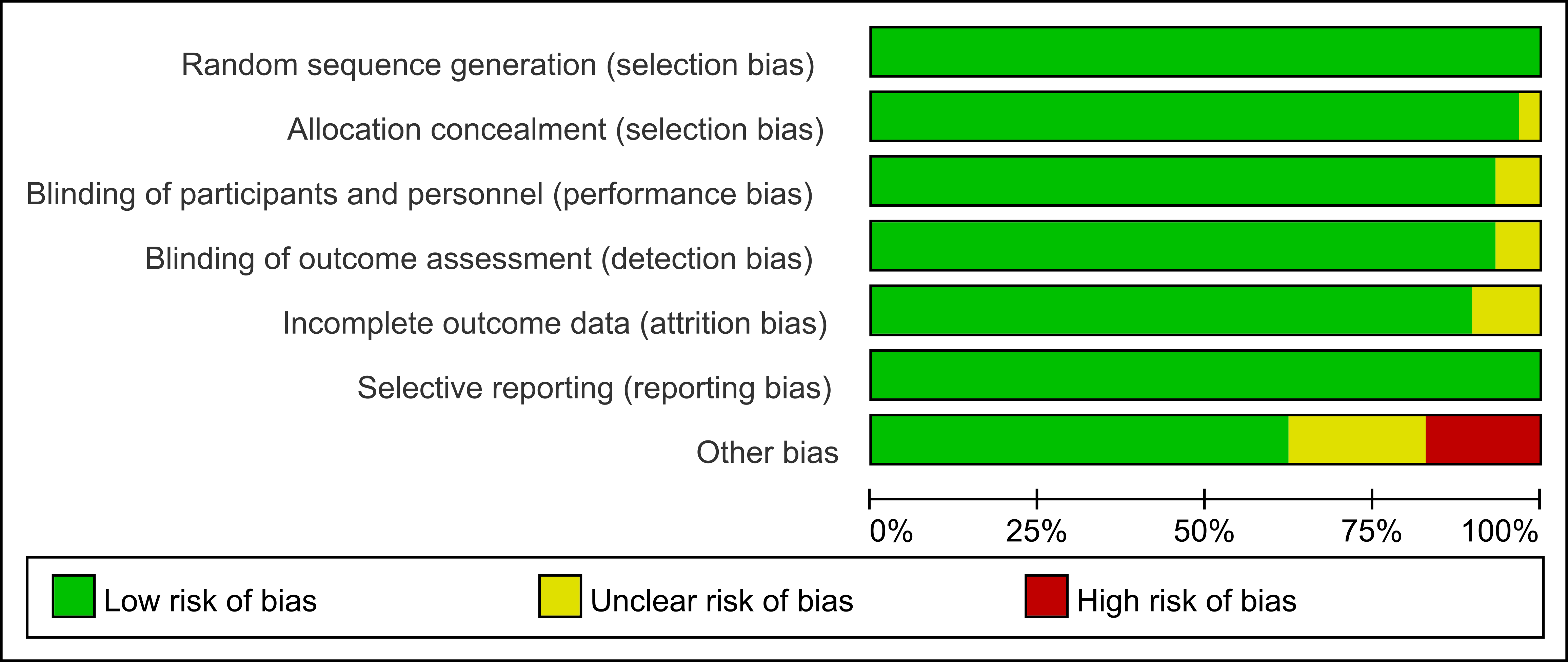

Supplement: Supplementary Figure 1 — Quality assessment of studies included in this meta-analysis (risk of bias graph). [file Image_1.TIF]

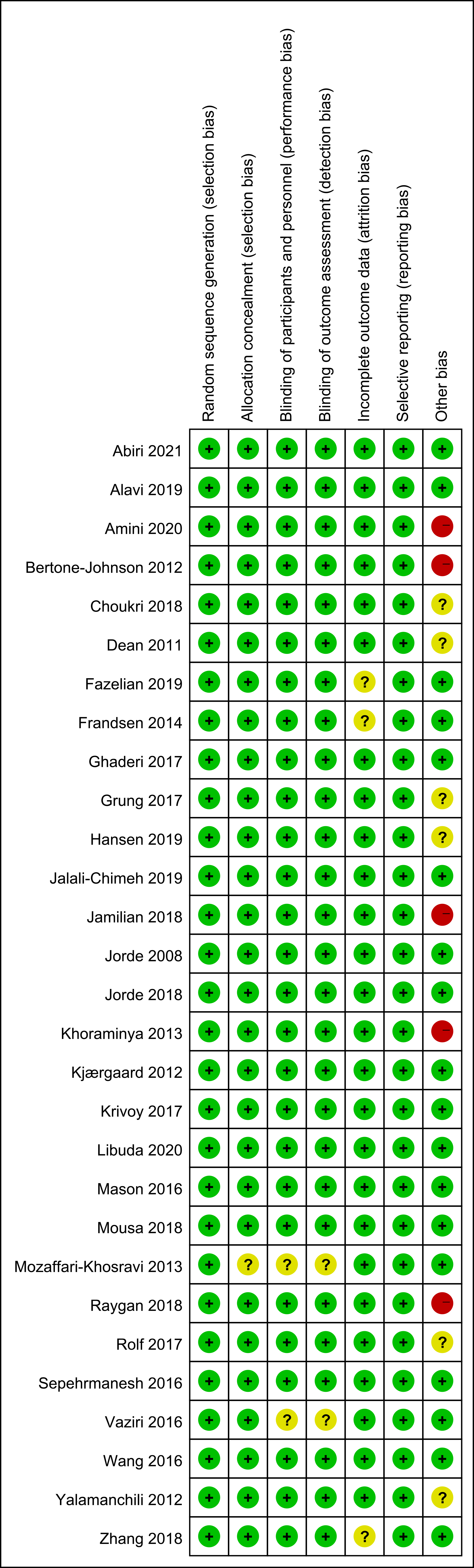

Supplement: Supplementary Figure 2 — Quality assessment of studies included in this meta-analysis (Risk of bias summary). [file Image_2.TIF]

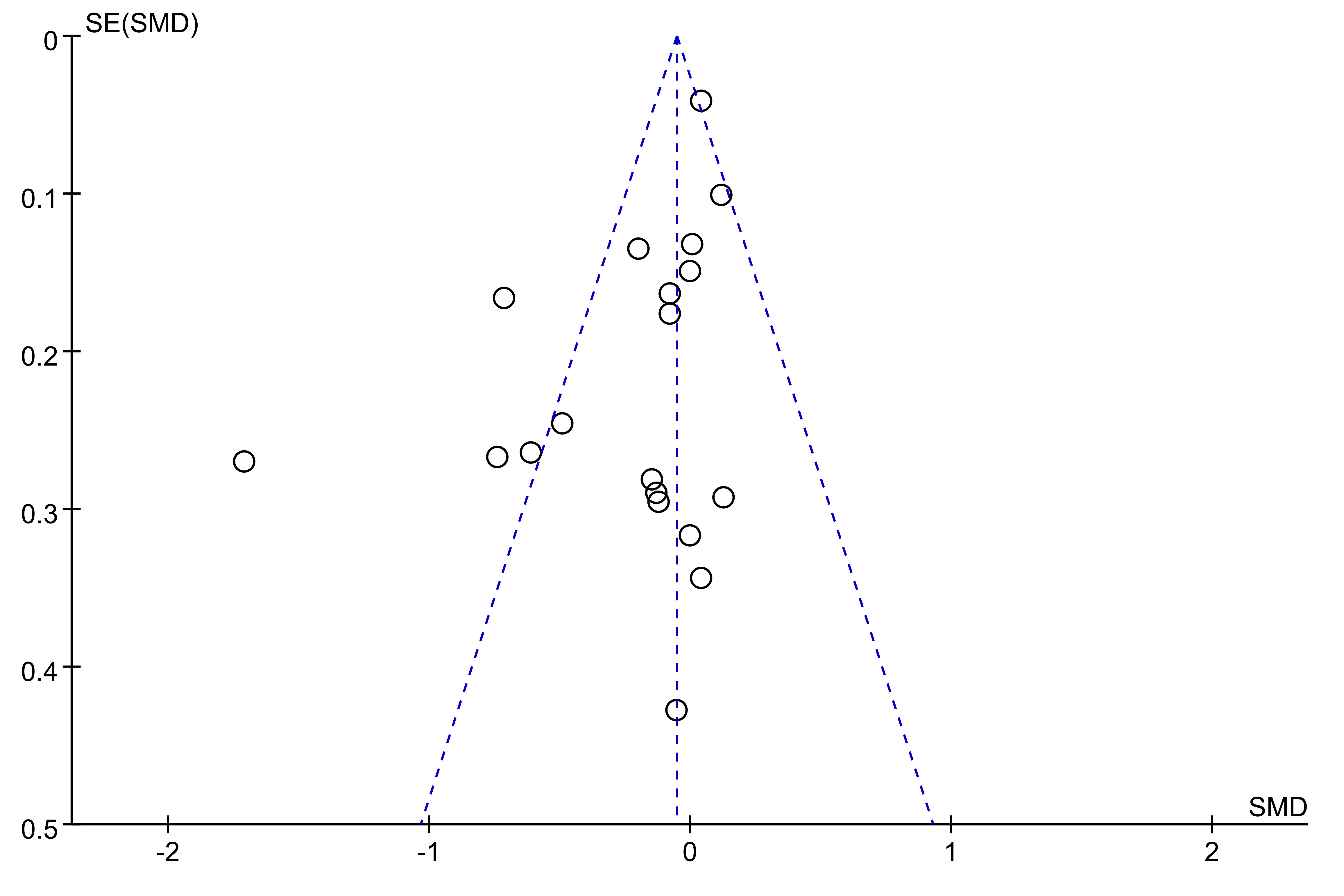

Supplement: Supplementary Figure 3 — Funnel plot for publication bias (the correlation between vitamin D and the incidence of depression). [file Image_3.TIF]

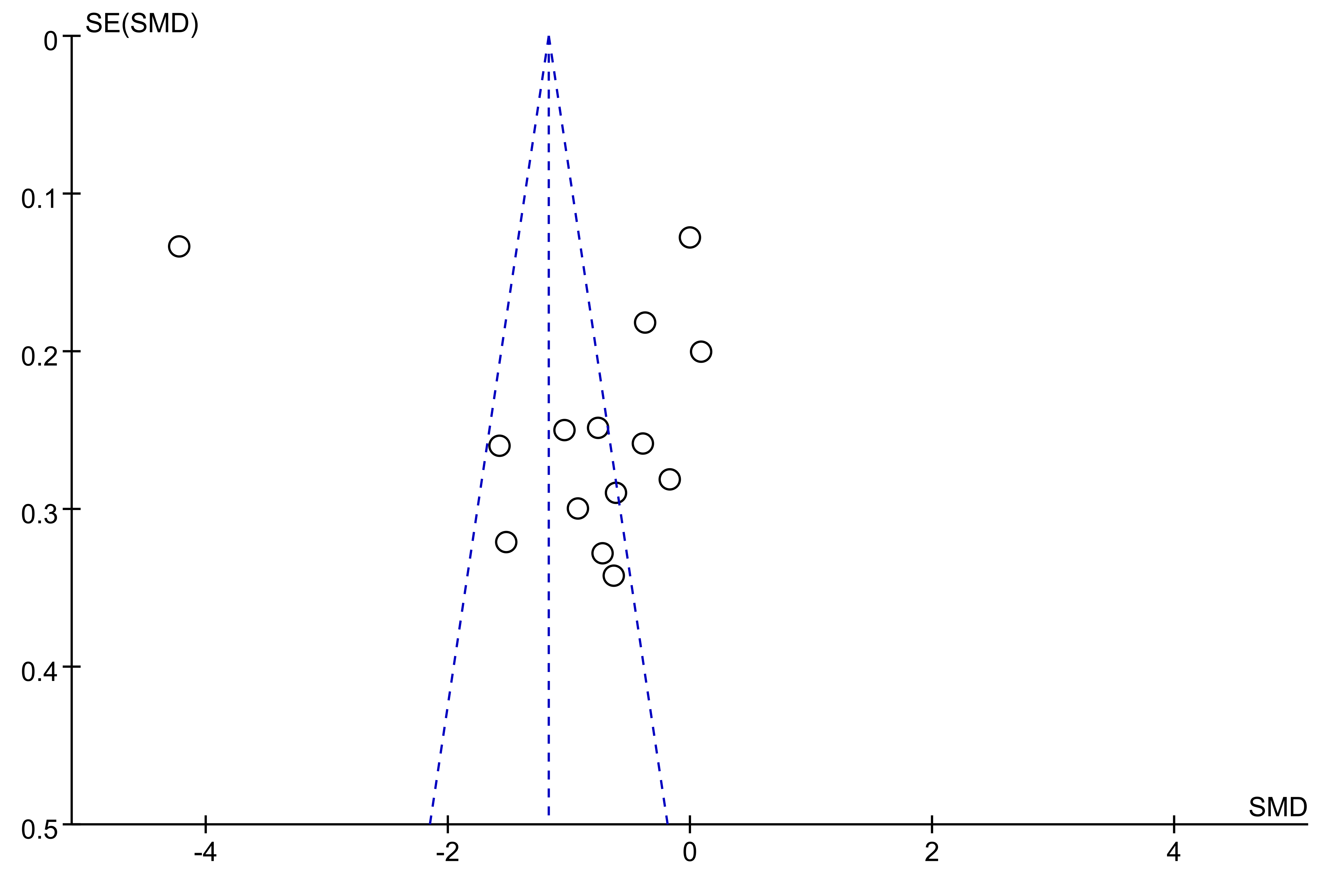

Supplement: Supplementary Figure 4 — Funnel plot for publication bias (the correlation between vitamin D and the prognosis of depression). [file Image_4.TIF]
